# Nuclear Transfer Perturbs Genomic Balance

**DOI:** 10.3390/epigenomes10020038

**Published:** 2026-06-05

**Authors:** Eryk Andreas, Justin C. St John

**Affiliations:** Experimental Mitochondrial Genetics Group, School of Biomedicine, The University of Adelaide, Adelaide, SA 5005, Australia; erykandreas@gmail.com

**Keywords:** mtDNA, oocyte, nuclear transfer, metaphase II spindle transfer, mitochondrial supplementation, gene expression, genomic balance, preimplantation embryo

## Abstract

Background: The transfer of a nucleus from one oocyte to another offers patients harbouring high levels of mitochondrial DNA mutation and sufferers of frequent fertilisation failure or early embryonic arrest the potential to have healthy children. However, a small amount of mtDNA is carried over with the nucleus as the transfer takes place. Consequently, we still need to distinguish between the effects of the carryover and the transfer of a nucleus itself from a mature oocyte. Methods: To overcome this, we analysed a series of hatching stage blastocysts generated using metaphase II spindle transfer and mitochondrial supplementation. The latter approach also introduces a small amount of mtDNA into the oocyte as fertilisation takes place. For both manipulations, an autologous approach was used to overcome the effects of third-party transfer. Results: We then compared the changes in global gene expression between the two groups. We found that the nuclear transfer process affected a number of gene networks and pathways. These included metabolic, cell cycle, inflammatory and immune, and epigenetic responses. A comparison with earlier stage blastocysts did not suggest that the cause was due to developmental delay. Conclusions: Critically, these changes could affect offspring health and well-being as is the case following somatic cell nuclear transfer.

## 1. Introduction

It is becoming increasingly evident that the nuclear and mitochondrial genomes need to communicate to enable effective cellular function. As a result, their interactions require modifications at any stage of a cell’s lifetime, a concept described as ‘genomic balance’ [1]. This is especially important throughout development when there are changes to the epigenome that regulate the expression of genes from the nuclear genome along with concomitant changes in mitochondrial DNA (mtDNA) copy number. This is highly apparent during oogenesis when the primordial germ cells, the precursor germ cells [2], initiate differentiation. This process is completed when the metaphase II (MII) stage is reached and oocytes have fertilisation potential. As a result, the highly DNA methylated primordial germ cells are subjected to a series of global DNA demethylation events mediated by TET-hydroxylation [3,4] until they reach the germinal vesicle stage when CpG methylation is reset. At the same time, mtDNA copy increases from 1400 [5] to ~300,000 [6] copies. CpG methylation then remains stable up to the MII stage [7] with mtDNA copy number being refined to >250,000 copies [6]. These processes lay the foundation for epigenetic inheritance from a maternal perspective [2] and the maternal-only inheritance of mtDNA [8].

Throughout preimplantation development, both sperm- and oocyte-driven methylation set the embryo’s methylome with at least three waves of DNA methylation prior to and including the blastocyst stage of development [7]. A key event is embryonic genome activation. This takes place between the 4- and 8-cell stages in larger mammals, namely, pigs and cattle. Similar patterns of DNA methylation are present in humans during early development [7]. Synchronously, there are changes in mtDNA copy number. Apart from a minor turnover event prior to the 4-cell stage, mtDNA copy number is reduced throughout development of the preimplantation embryo until the late morula/early blastocyst stage. At this stage, mtDNA replication commences [6,9]. However, it is restricted to the trophectodermal cells [9], which are the precursors of the placenta. The inner cell mass cells continue to dilute their mtDNA copy number until post-gastrulation. Replication of mtDNA then proceeds in a cell-specific manner. Nevertheless, the three waves of DNA methylation are complete by the late blastocyst stage [7].

Some of the more invasive assisted reproductive technologies are either being introduced into clinical medicine or are being investigated in readiness for implementation. However, they can distort the patterns of epigenetic and mtDNA inheritance [10,11]. Some of these technologies originate out of somatic cell nuclear transfer [12,13], for example metaphase II spindle transfer (MST) and pronuclear transfer (PNT) [14,15]. They have been proposed to help carriers of mtDNA disease or patients with repeated fertilisation or embryonic developmental failure to have children [16]. However, they are associated with delays in DNA methylation during preimplantation development [10] and altered gene expression profiles [11]. Consequently, the transfer of nuclei between oocytes or zygotes will necessitate the nucleus or nuclei to acclimatise to its/their new cytoplasmic environment. They will also need to interact with the resident cohort of mitochondrial genomes. This series of complex processes could lead to aberrant patterns of gene expression [11] or developmental delay [10]. To this end, we have shown that there were significant differences in gene expression and pathways between hatching blastocysts generated through MST and oocytes fertilised by intracytoplasmic sperm injection (ICSI) [11].

A confounding situation with the nuclear transfer technologies is that a small amount of mtDNA accompanies the nucleus as the somatic cell [17], spindle [18,19] or pronuclei [20,21] are transferred into the recipient oocyte or zygote, respectively. Indeed, it is evident that the introduction of a few copies of carried over mtDNA can alter the DNA methylation and gene expression patterns of blastocyst stage embryos [22]. This has been demonstrated in another assisted reproductive technology, namely mitochondrial supplementation as ICSI is performed (mICSI). Furthermore, it impacts on the DNA methylation and gene expression patterns in the tissues of resultant offspring [23]. Importantly, this issue has yet to be resolved for MST and PNT and results in offspring inheriting variable rates of carried over mtDNA [19,20]. Consequently, as two genomes are at play, it is difficult to determine which effects relate to the mitochondrial genome and which relate to the process of nuclear transfer.

The pig is an excellent model for the study of human development. A number of its organ systems and responses to physiological and pathophysiological stimuli are similar to those of humans [24,25]. Likewise, the pig’s embryology and early development are also representative of those in the human [3,7]. Here, we have used our pig model to analyse two sets of blastocyst stage embryos that have reached the hatching stage and are thus primed for implantation. The first was previously generated through MST where the MII spindle was removed and returned to its cytoplasm and, thus, possessed a small amount of mtDNA carryover and then fertilised by ICSI [11]. The second set was generated through mICSI where, again, a small amount of mtDNA was introduced into the oocyte as it was fertilised. Consequently, both sets of embryos possessed mtDNA introduced into the mature oocyte and were fertilised by ICSI. This, then, allowed us to focus specifically on the nuclear transfer procedure itself without the confounding complication of the carried over mtDNA. In other words, this allowed us to determine how the nuclear transfer process alone affects early global gene expression through RNA-seq and bioinformatics analyses. We further determined whether hatching blastocysts derived through MST exhibited developmental delay by analysing their global gene expression profiles with expanded blastocysts derived through mICSI. We report that nuclear transfer alone affects a large cohort of genes and a number of key developmental pathways, but they are more equivalent to mICSI-derived hatching blastocysts than their expanded counterparts.

## 2. Results

### 2.1. Comparison of MST with mICSI at the Hatching Blastocyst Stage

**Figure 1:**
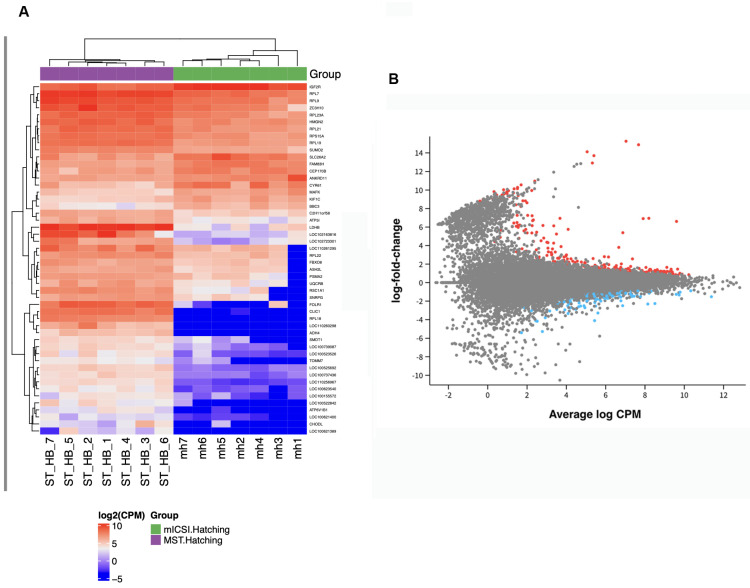
Differential gene expression between MST- (*n* = 7) and mICSI-derived (*n* = 7) hatching blastocysts. Heat map representation of the top 50 most differentially expressed genes highlighting the clustering of each hatching blastocyst. A statistical cut off point of FDR < 0.05 was employed (**A**). Graphical representation of the distribution of genes expressed represented by log fold change (**B**). In each case, red indicates genes significantly upregulated and blue indicates those downregulated. Grey indicates presence of all other genes. CPM = counts per million.

To determine how nuclear transfer alone affects global gene expression, we compared the gene expression profiles of hatching blastocysts generated through MST (MST-H) with those derived through mICSI (mICSI-H). We chose the hatching blastocyst stage as the three waves of DNA methylation would be complete by then and these embryos have implantation potential. In all, 423 gene transcripts (342 identifiable genes) were differentially expressed at FDR < 0.05 (Figure 1; Supplementary Table S1). The top 20 genes and their functions are listed in Table 1 and included genes associated with ribosomal proteins, metabolism, cell cycle and epigenetic regulation. We then performed KEGG pathway analysis and identified 32 affected pathways at *p* < 0.05 (Supplementary Table S2). The top 10 affected pathways (Table 2) included Ribosome-, Coronavirus-, Oxidative phosphorylation-, Endocytosis- and Oocyte meiosis-associated pathways. We then focused on pathways specifically upregulated (Table 2 and Table S3) and downregulated (Table 2 and Table S4). For the upregulated pathways, the five above-mentioned pathways were all upregulated whilst for the downregulated pathways those associated with inflammation (TNF signalling); metabolism (AMPK signalling); cells’ responses to stress, e.g., DNA damage (p53 signalling); and cell growth, proliferation and survival (PI3K-Akt signalling) were affected. The discordant expression of genes associated with Oxidative phosphorylation, metabolic signalling, cell growth and proliferation, and immune responses represent disruption to key early developmental events. This, in turn, suggests that the interactions between the nuclear and mitochondrial genomes were not fully reestablished.

We then analysed the RNA-seq data outputs in Reactome to determine if they had any significance to human outcomes and, specifically, disease (Table 3 and Table S5). Pathways and interactions associated with immune responses (interferon activity), transcription, cell growth and proliferation were all upregulated suggesting that there was a ‘recognition’ response to the transferred nucleus and that cell cycle regulation, growth and transcription were also affected.

### 2.2. Comparison of MST-H with mICSI-Derived Expanded (mICSI-E) Blastocysts

**Figure 2:**
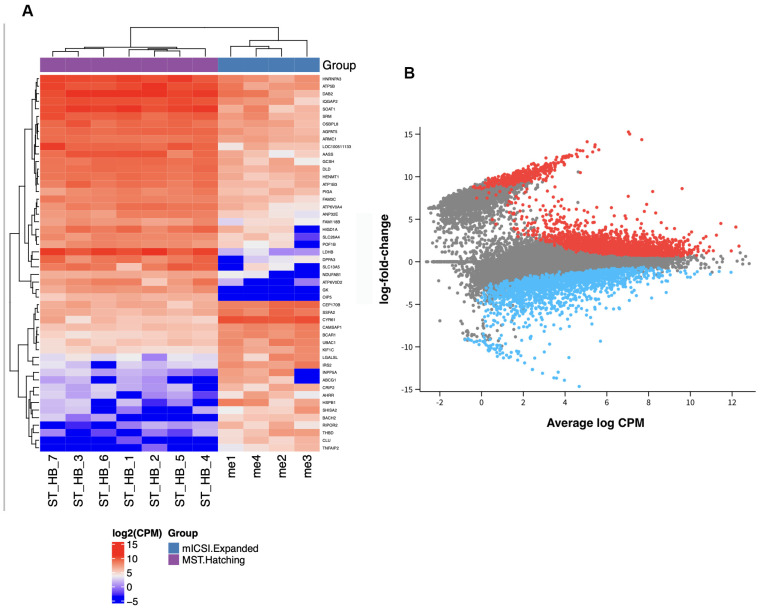
A comparison of the differential gene expression between MST-derived hatching blastocysts (*n* = 7) and mICSI-derived (*n* = 4) expanded blastocysts. Heat map highlighting the top 50 differentially expressed genes and the clustering of each blastocyst (FDR < 0.05) (**A**). The distribution of expressed genes represented as log fold change (**B**). Red highlights genes significantly upregulated and blue downregulated. Grey dots indicate all other genes. CPM = counts per million.

In order to ascertain if differential gene expression was due to developmental delay resulting from the process of nuclear transfer, as observed in somatic cell nuclear transfer [26], we compared the gene expression profiles of MST-H blastocysts with mICSI-E blastocysts. A total of 3937 genes were differentially expressed (3423 identifiable genes) (FDR < 0.05) (Figure 2; Supplementary Table S6) with the top 20 genes and their respective functions listed in Table 4. Again, this gene list comprised those associated with metabolism, epigenetic regulation, Ribosomes, cell cycle and inflammation. It also included factors associated with pluripotency. All the differentially expressed genes were assigned to 45 pathways when assessed by KEGG (Supplementary Table S2). Amongst the top 10 pathways were Oxidative phosphorylation, cell cycle, Cellular senescence, and Citrate cycle (TCA cycle) (Table 5). Those pathways that were upregulated included Oxidative phosphorylation, Metabolic pathways, cell cycle, Citrate cycle (TCA cycle), and DNA replication (Table 5 and Table S3) whilst the downregulated pathways included PI3K-Akt signalling, ECM-receptor interaction, signalling pathways regulating pluripotency of stem cells, and MAPK signalling (Table 5 and Table S4). Reactome pathway analysis again highlighted pathways associated with immune and inflammatory responses, metabolism, DNA synthesis and epigenetic regulation (Table 6 and Table S7). The larger number of differentially expressed genes between the MST-H and mICSI-E blastocysts suggest that the differences are largely due to developmental differences rather than the nuclear transfer process *per se*. Nevertheless, there were some similar differences that were observed in the MST-H and mICSI-H comparison. This included pathways and networks associated with Oxidative phosphorylation, cell cycle and immune responses.

A comparison of the commonly differentially expressed genes between the MST-H versus mICSI-H blastocysts and MST-H versus mICSI-E blastocysts revealed that 123 genes were uniquely expressed in the MST-H versus mICSI-H blastocyst comparison (Supplementary Table S8) and 3637 genes uniquely expressed in the MST-H versus mICSI-E blastocyst comparison (Supplementary Table S8). Conversely, a smaller number of differentially expressed genes were common to the two comparisons. When these two gene lists were analysed in Reactome, pathways and interactors associated with ribosomal activity and translation (Supplementary Table S9) and defective homologous recombination and cell cycle were affected (Supplementary Table S10), respectively. Consequently, there were fewer unique genes differentially expressed in the MST-H versus mICSI-H blastocyst comparison but a greater number of affected pathways and interactors.

## 3. Discussion

During oogenesis a number of genomic, epigenomic and cellular processes are established that enable a primordial germ cell to mature into a MII oocyte. These involve, for example, waves of DNA methylation/demethylation to the nuclear genome [5,7] and exponential replication and subsequent refinement of mtDNA copy number [6,27,28]. As a result, levels of maternal DNA methylation and mtDNA are set prior to fertilisation. Post-fertilisation, requisite levels of maternal and paternal DNA methylation are established by the late blastocyst stage [7]. In addition, mtDNA has been segregated to the two distinct cellular lineages that arise at this stage of development, namely the trophectoderm and in the inner cell mass [27]. We generated a series of hatching blastocysts through MST, as documented in [11], and mitochondrial supplementation, which both have introduced similar levels of mtDNA (350 to 780 copies) [11,22], and were fertilised by ICSI. This enabled us to determine the effects of the nuclear transfer process on gene expression in the mature oocyte at a key developmental stage in embryos with implantation potential. Consequently, as both sets of embryos were fertilised in a similar manner, we did not include a comparison with hatching blastocysts derived through non-manipulation. Indeed, our aim was to directly compare two sets of embryos where the only difference was the removal or otherwise of a MII spindle. In each case, autologous transfer was performed to circumvent a third-party effect at a nuclear DNA and mtDNA level [11] and the effects of ICSI on embryo development [29] were overcome. In this instance, autologous nuclear transfer refers to the removal of the spindle and its replacement within the same oocyte. This is distinct from a clinical protocol that would transfer the patient’s nucleus into a third-party oocyte.

By simply removing a MII spindle and returning it to its own cytoplasmic environment, we observed a host of genes that were differentially expressed by the hatching blastocyst stage. This suggests that the reconstructed oocyte is seeking to accommodate the returning nucleus and manage any damage that may have arisen through its extraction. The underlying question, then, is whether the process would cause a series of downstream events that could be harmful to the developing foetus and offspring or whether the effects would be benign. Importantly, we note the immune pathways were aberrantly regulated, as determined by KEGG and Reactome analyses. To this extent, the differentially regulated interferon responses are analogous to those associated with interactions with mtDNA [30] and resulting from cell stress [31].

In the case of mtDNA, it appears that the reintroduced nucleus is likely responding to the resident population of mtDNA in the oocyte rather than the very small population attached to the karyoplast. In essence, this suggests that the returning nucleus does not necessarily recognise the cytoplasm as being ‘self’ but rather a distinct entity, although it had previously interacted with this genome from the primordial germ cell stage onwards and carries a small population of mitochondria attached to its membrane. This may arise through the extraction process conditioning the attached mitochondria in a manner that causes the resident oocyte population to be deemed different. Nevertheless, the exposure, in our case, would be different to other forms of nuclear transfer where the karyoplast may undergo fusion, electrically or through virus, to an enucleated donor oocyte [32]. For cell stress, it appears that the mechanical processes of protrusion, extraction and replacement induce a sense of damage or, at least, significant change that instigates an immune chain response. Indeed, it has become increasingly evident that both the nuclear and mitochondrial genomes can elicit immune responses along with some of the epigenetic regulators [33]. For example, mitophagy, the process of mitochondrial autophagy, mediates the elimination of sperm mtDNA prior to embryonic genome activation in the newly formed embryo [34]. Likewise, more recently, it has been proposed as a mechanism for regulating the segregation of mtDNA at the zygote stage in mice whereby defective mtDNA are selected for elimination [35]. In our work, we identified mitophagy as an upregulated pathway following KEGG and Reactome analysis (Supplementary Tables S3 and S5). Furthermore, one factor associated with another form of paternal mtDNA regulation, namely, mitochondrial unfolded protein response (Supplementary Table S5), was also modulated. This has been associated with the accumulation of paternal mtDNA through an ALK-B1 mediated tRNA m1A epitranscriptomic modification [36].

Whilst cell growth and proliferation were downregulated, Oxidative phosphorylation was upregulated at the hatching blastocyst stage. These events are asynchronous with this stage of development. Cell proliferation would likely be upregulated in line with the need to generate sufficient cells to support the expansion of the embryo and transition to foetal development. Furthermore, cell proliferation normally utilises glycolysis or aerobic glycolysis as the predominant form of energy production [37]. This is a more efficient form of energy production as it provides lower levels of energy at a faster rate to support cell cycle activity and protein production [38]. On the other hand, cell growth is required to support the expansion of the trophectoderm to support the invasive trophoblasts that will give rise to the placenta [39], which in turn are reliant on growth factors modulated by innate and adaptive immune regulators [40].

In terms of the regulation of early development, we identified modulation of factors associated with the citric acid cycle, a metabolic pathway also present in the mitochondrion. The citric acid cycle donates electrons to the electron transfer chain to promote the production of ATP derived from Oxidative phosphorylation [38]. Nevertheless, by-products of the citric acid cycle are also mediators of DNA methylation. In this respect, they act as co-factors in the conversion of methylated DNA, 5-methylcytosine (5-mC), to its transient state, 5-hydroxy-methylcytosine (5-hmC) [41]. This process is key to the resetting of the embryonic genome during human preimplantation development [42] with depletion of 5-hmC affecting the activation of embryonic genes [43]. Indeed, other epigenetic regulators were also identified as being aberrantly regulated including histone lysine methyltransferase complex subunit ASH2-L and concomitant upregulation of a number of transcription factor pathways such as TFAP2 and RUNX1.

Our comparison of MST hatching blastocysts with mICSI expanded blastocysts highlighted a large number of differentially expressed genes including metabolic, steroidogenic and epigenetic regulators. However, the number was larger than for the comparison between the two hatching blastocyst cohorts, suggesting that the MST-derived hatching blastocysts were closer to their mICSI-derived counterparts. This indicates that the larger differences observed were a result of differences in stages of development rather developmental delays. Nevertheless, the aberrant patterns of reprogramming through the expression of epigenetic regulators, metabolism and cell cycle suggest an overlap and similarity to blastocysts derived through SCNT [26].

Whilst our autologous approach to nuclear transfer in this work would not be used in the clinical setting, it has clinical context. It clearly shows that the process of nuclear transfer through MST, and most likely PNT also, is mechanically disruptive to the oocyte and results in aberrant gene expression profiles. As a result, these anomalies could have implications for offspring health and well-being. From the limited number of large animals, primarily rhesus macaques, generated through MST, there is no evidence of serious abnormalities [18]. Nevertheless, insufficient, clinically relevant large animals have been generated or analysed to provide sufficient reassurance. Given that we observed errant metabolic, epigenetic and immune response pathways at the hatching blastocyst stage, it is important to establish whether associated disorders such as autoimmune, epigenetic or metabolic disorders would affect offspring. Indeed, from SCNT studies in large animal models [44,45], a large number of abnormalities have been reported that result from incomplete reprogramming and failure of the nucleus to adapt to its new cytoplasmic environment. These include birth-related defects, increased levels of post-natal loss, dysfunction of the immune system, failure of the kidneys and heart to function effectively, large offspring syndrome, circulatory-associated disorders, distress within the respiratory system, and movement and balance disorders, suggesting neurological dysfunction. These disorders add to the range of disorders that could occur when mtDNA carryover harbouring mutant mtDNA is selected for and potentially results in severe or even fatal mtDNA diseases [46]. Even the addition of extra copies of mtDNA introduced through mICSI can result in aberrant DNA methylation and gene expression profiles at the blastocyst stage [22]. Some of these effects are transmitted through to the offspring resulting in differential DNA methylation and gene expression profiles in their tissues [47]. It also leads to some key developmental milestones related to growth and biochemical and haematological parameters not being met or their delay in being established, as determined in offspring [23].

Perhaps Germinal Vesicle Transfer offers an opportunity to offset some of the epigenetic changes as the transfer of an earlier stage nucleus would allow time for its acclimatization to the ‘foreign’ cytoplasm. The maturing oocyte would then proceed through germinal vesicle breakdown and, following in vitro maturation, progress to MII in readiness for fertilisation [48]. However, a modified approach, namely ‘aggregated chromosomes transfer’, would allow similar processes to take place but considerably reduce the potential for mtDNA carryover [49]. Indeed, Australian reproductive law has made provision for further research to be conducted into Germinal Vesicle Transfer as an approach to overcome the transmission of mutant or deleted mtDNA from a carrier [50].

In conclusion, we have analysed a cohort of final stage preimplantation embryos that highlight the direct effects of the nuclear transfer approach through MST. By design, we have been able to exclude the effect of mtDNA carryover as shown in other models [22,23] and the effects of the fertilisation procedure (ICSI). Our data point to a large number of genes that are affected by nuclear transfer alone and influence key developmental pathways. However, these embryos appear to be nearer to their mICSI-counterparts than the earlier staged mICSI-expanded blastocysts. Nevertheless, there is commonality with SCNT-derived embryos suggesting that the removal and return of a nucleus alone induces a series of metabolic, cell cycle, inflammatory and immune, and epigenetic responses that could have downstream implications for offspring derived through such approaches. Consequently, it is imperative that a series of full-scale investigations are conducted in large animal models clinically relevant to humans before the nuclear transfer technologies undergo further clinical trialling or implementation.

## 4. Materials and Methods

### 4.1. Ethics Statement

The use of porcine oocytes and ovaries to generate embryos was registered with the University of Adelaide’s Animal Ethics Committee. However, formal ethics approval was not necessary as the material was collected from an abattoir after the material had been sourced for food production. As a result, no live animals were used and no animals were killed for research purposes.

All reagents and chemicals were purchased from Sigma-Aldrich (Bayswater, VIC, Australia) unless otherwise mentioned.

### 4.2. Oocyte Collection and In Vitro Maturation

Pairs of ovaries from gilts were obtained from a local abattoir, retained as pairs and transported to the laboratory in 0.9% NaCl solution (Baxter, Old Toongabbie, NSW, Australia) and maintained at 38.5 °C. Cumulus-oocyte-complexes (COCs) exhibiting diameters of 3–6 mm were aspirated from follicles of each pair of ovaries using an 18 G needle. Following isolation, the COCs from each pair were washed three times in handling media (medium 199-Hepes; (Thermo Fisher, Waltham, MA, USA)) which was supplemented with 10% filtered gilt follicular fluid (FF) and 5 µg/mL cycloheximide (CHX). They were then cultured in individual wells containing 500 μL CHX-supplemented in vitro maturation (IVM) media at 38.5 °C in a humidified incubator at 5% CO_2_ in air for 18–20 h. The IVM media comprised medium 199 (Thermo Fisher) supplemented with 0.88 M cysteamine, 5 μg/mL insulin, 0.61 mM L-glutamine, 0.80 mM Na-pyruvate, 10 IU/mL Folligon^®^ (Pacific Vet Pty Ltd., Braeside, VIC, Australia), 10 IU/mL Chorulon^®^ (Pacific Vet Pty Ltd.), and 0.10 μg/mL epidermal growth factor (EGF). COCs were then washed three times in IVM media minus CHX and cultured for another 25 h in IVM media minus CHX at 38.5 °C 5% CO_2_.

### 4.3. Collection of MII Oocytes

For the collection of MII oocytes, each individual well of 500 µL of expanded COCs received 10 µL of 25 mg/mL hyaluronidase to aid removal of the cumulus cells. The COCs were transferred to handling media for complete denudation using a narrow glass pipette. MII oocytes were then selected based on the presence of a polar body and retained in a 50 µL drop of handling media prior to microinjection. Additionally, only MII oocytes with a clear perivitelline space and a distinct separation between the oolemma and zona pellucida were used; otherwise, the oocytes were prone to lysis during enucleation.

### 4.4. MST

Just before the transfer of the spindle, MII oocytes were cultured for 5 min with 5 µg/mL cytochalasin B in handling media. Oocytes were then placed into individual 3 µL droplets comprising Porcine X media (PXM, Supplementary Table S11). Each oocyte was held by a holding pipette (H-30/120-30, ICSION Medical Pty. Ltd., Thebarton, SA, Australia) and its spindle observed via Oosight (Cambridge Research Instruments, Cambridge, UK). The spindle was then oriented to the 2–4 o’clock position. The spindle surrounded by minimal cytoplasm was aspirated with a 11 µm spike ICSI pipette (I-11-30, ICSION Medical Pty. Ltd.) under the Oosight filter. A 11 µm diameter spiked pipette minimises disruption to the metaphase plate, reduces damage to the chromosomes, and minimises mtDNA carryover of the accompanying cytoplasm [11]. Excess cytoplasm is further removed by expulsion of the spindle into the culture media followed by re-aspiration to reduce mtDNA carryover to 306 ± 49 copies [11]. Following enucleation, the spindle was returned to its original cytoplasm through the same break in the zona pellucida. Reconstructed oocytes were collected in a droplet of handling media.

### 4.5. Fertilisation

Spermatozoa in semen extender were provided by Terminal Mix (Sabor Ltd., Clare, SA, Australia). Initially, the extender underwent centrifugation at 300× *g* for 5 min and the supernatant was discarded. The spermatozoa were washed in sperm preincubation media (SPM) that had been pre-equilibrated and comprised medium 199 additionally containing 0.91 mM sodium pyruvate, 4.1 mM calcium lactate, 75.0 µg/mL streptomycin sulphate, 37.5 µg/mL penicillin-G, and 12% heat inactivated foetal bovine serum (Thermo Fisher); equilibrated for 4 h at 38.5 °C 5% CO_2_, and cooled to room temperature prior to the sperm pellet being resuspended. Spermatozoa were then centrifuged at 300× *g* for 5 min, resuspended in SPM and retained until use in the dark at room temperature.

Approximately 30 min prior to fertilisation, ICSI dishes were prepared with SpermSlow™ (Cooper Surgical Inc., Sydney, NSW, Australia), as described in the manufacturer’s instructions. To this extent, 2 × 10 µL droplets of SpermSlow™ and 5 µL droplets of handling media were loaded onto the lid of a 60 mm culture dish (Thermo Fisher). 1–2 µL of the sperm drop were added to the right of the leftmost SpermSlow™ drop and the two droplets merged. The droplets were covered with SAGE mineral oil (Cooper Surgical Inc.) and incubated at 38.5 °C 5% CO_2_ for 15 min. ICSI was performed with a 6 µm pipette (I-6.0-30; ICSION Medical Pty. Ltd.) and active spermatozoa selected. A single spermatozoon was mechanically immobilised and collected in the ICSI pipette containing SpermSlow™. Each reconstructed oocyte was clasped by a holding pipette with the reconstructed karyoplast positioned at 12 o’clock relative to the polar body. The ICSI pipette entered at 3 o’clock and the spermatozoon released in the direction of the 6 o’clock position to prevent injury to the karyoplast.

### 4.6. Isolation of Oocyte Mitochondria for mICSI

Fractions containing mitochondria were isolated from non-MII oocytes, as described in [22,23]. Non-MII oocytes were classified as those oocytes failing to mature after in vitro maturation. This is indicated by the absence of the first polar body during MII oocyte assessment. In all, 10 denuded non-MII oocytes from each ovary pair were washed in 0.1% PVP and placed in 5 mL of mitochondrial isolation buffer (20 mM Hepes pH 7.6, 220 mM mannitol, 70 mM sucrose, 1 mM EDTA) containing 2 mg/mL BSA. After 5 min, the oocytes underwent homogenization of 10 strokes with a Potter–Elvehjem tissue grinder set (VWR International, Radnor, PA, USA). The homogenate was then centrifuged at 800× *g* for 10 min and approximately 2.2 mL was transferred into two 1.7 mL Eppendorf tubes and centrifuged at 10,000× *g* for 20 min. The mitochondrial pellets from both tubes were resuspended in 1 mL of mitochondrial isolation buffer and centrifuged at 10,000× *g* for 20 min. The resultant mitochondrial pellet was reconstituted in 2 µL of mitochondrial isolation buffer prior to supplementing MII oocytes at the time of mICSI.

### 4.7. mICSI

After picking up the sperm, as described above for ICSI, 5 pl of mitochondrial isolate, which contained circa 780 copies of mtDNA [51], were drawn into the pipette and injected into an MII oocyte from the same ovary pair.

### 4.8. Embryo Culture

Reconstructed oocytes were cultured in porcine zygote media (PZM, Supplementary Table S11) at 38.5 °C 5% CO_2_, 5% O_2_. After 48 h (Day 2) and 120 h (Day 5), putative embryos were placed in fresh media. Once blastocysts reached the expanded or hatching blastocyst stage (Day 7 or 8), they were washed in sterile PBS without Ca^2+^ or Mg^2+^, collected and frozen individually in 2 µL PBS at −80° C in readiness for further analysis.

### 4.9. RNA Extraction, Library Preparation and Sequencing

The Arcturus PicoPure RNA Isolation Kit (Thermo Fisher) was used to extract RNA from hatching- and expanded-stage blastocysts, as described in [52,53]. This generated between 1 and 18 ng of input RNA. Once extracted, RNA was stored at −80 °C. The Australian Genome Research Facility undertook preparation of libraries using the whole extract and the Illumina SMART-Seq Ultra Low Input Kit (Illumina, San Diego, CA, USA), as described by the manufacturer, along with 16 cycles of amplification and ribosomal RNA depletion using Illumina Ribo-Zero Gold (Illumina). They also sequenced the RNA on a NovaSeq X Plus (Illumina) applying 150 bp paired-end chemistry.

### 4.10. RNAseq Data Analysis and Identification of Differentially Expressed Genes

The primary sequence data were produced with the Illumina DRAGEN BCL Convert 07.031.732.4.3.6 pipeline. They were then inspected for Illumina adapters, over-representation of sequences and contamination from other species. In all, this generated between 51 million and 109 million reads. The sequenced reads were then mapped to the *Sus scrofa* genome (GCF_000003025.6) with the STAR aligner V2.3.5a [54]. The transcripts were assembled with the StringTie Tool v2.1.4 by applying the reference annotation-based assembly option. As a result, 64 to 89% of the reads mapped to the reference genome. edgeR (version 4.2.2) was used to identify differentially expressed genes (https://bioconductor.org/packages/release/bioc/html/edgeR.html) using R 4.4.0 (accessed on 17 October–11 November 2025).

### 4.11. Functional Pathway Enrichment and Gene Network Analysis

Differentially expressed genes underwent KEGG pathway [55] over-representation analysis in edgeR. Reactome pathway analysis was performed using reactome.org and the gene analysis tool, PADOG (accessed 18–19 December 2025) [56]. Reactome analysis was carried out using official gene symbols and non-human identifiers projected to human. Pathways at *p* < 0.05 were regarded to be over-represented as a function of the number of genes present in each pathway for the KEGG analyses. Reactome pathways with an FDR of <0.05 were deemed to be over-represented in relation to the number of genes present in a given pathway. For KEGG, over-representation analyses included separate analyses of up- and downregulated genes. This approach provides the best statistical power for determining biologically significant pathways [57]. Upregulated and downregulated pathways were also combined in one table for the KEGG analyses to determine the overall total number of significant pathways.

### 4.12. Statistical Analysis

For differential gene expression analyses, the Bioconductor package edgeR v4.2.2 [58] was used within R v4.4.0 [59]. Within edgeR, the counts between blastocysts were normalised using the default trimmed mean of M values normalisation method. Differential expression between the groups was quantified using a generalised linear model to produce *p*-values. The false discovery rate (FDR) was generated by edgeR using the Benjamini–Hochberg method. FDR < 0.05 indicated over-representation for the number of genes identified in a given pathway.

## Figures and Tables

**Table 1:** Top 20 differentially expressed genes as a result of nuclear transfer through the comparison of MST hatching blastocysts with mICSI hatching blastocysts. The full list of genes is available in Supplementary Table S1. Genes highlighted in red are associated with the preimplantation embryo.

Gene ID	Gene or Human Equivalent	Function
LDHB	Lactate dehydrogenase B	Catalyses the interconversion of pyruvate and lactate
CLIC1	Chloride Intracellular Channel 1	Stabilisation of cell membrane potential
RPL18	Ribosomal Protein L18	Catalyses protein synthesis
FOLR1	Folate Receptor Alpha	Linked to membranes through glycosyl-phosphatidylinositol or exists as a soluble
LOC100525692	60S ribosomal protein L7a	Interacts with nuclear hormone receptors, e.g., thyroid hormone receptor
LOC102163816	MALAT1	Mediates cell cycle transition from G1 to S
LOC100737436	Protein S100-A16 pseudogene	Pseudogene
LOC102723301	RNA, 7SL, cytoplasmic 1	Helps coordinate a global cellular response to stress
LOC110260298	Unspecified	
RSC1A1	Regulator Of Solute Carriers 1	Inhibits solute carrier family 5 (sodium/glucose cotransporter), member 1 gene (SLC5A1) expression and reduces SLC5A1 exocytosis
LOC100739087	Large ribosomal subunit protein eL14	Large ribonucleoprotein complex undertaking protein synthesis
LOC110256967	40S ribosomal protein S17 pseudogene	Pseudogene
RPL19	Ribosomal protein L19	Catalyses protein synthesis
ASH2L	ASH2 like, histone lysine methyltransferase complex subunit	Enables binding of beta-catenin activity and cis-regulatory region transcription. Regulates cell proliferation, stimulated by oestrogen, and involved in chromatin remodelling. Modulated by DNA damage
UQCRB	Ubiquinol-cytochrome c reductase binding protein	Binds to ubiquinone and is involved in electron transfer
FAM83H	family with sequence similarity 83 member H	Develops structure and calcification of tooth enamel
CYR61	cysteine rich angiogenic inducer 61	Inducer of growth factor
SNRPG	Small nuclear ribonucleoprotein polypeptide G	A component of the U1, U2, U4, and U5 small nuclear ribonucleoprotein complexes, precursors of the spliceosome. The protein may also contribute to the U7 small nuclear ribonucleoprotein complex, which is involved in 3′ end of histone transcript processing.
ADH4	Alcohol dehydrogenase 4 (class II)	Metabolises products associated with ethanol, retinol, other aliphatic alcohols, hydroxysteroids, and lipid peroxidation.
LOC100523526	40S ribosomal protein S23 pseudogene	Pseudogene

**Table 2:** KEGG pathway analysis for differentially expressed genes from the comparison between MST-H and mICSI-H blastocysts. Only the top 10 pathways are shown. The full list is available in Supplementary Tables S2–S4. Pathways highlighted in red are associated with the preimplantation embryo.

MST-H vs. mICSI-H (Overall)	MST-H vs. mICSI-H (Upregulated)	MST-H vs. mICSI-H (Downregulated)
Ribosome	Ribosome	Longevity regulating pathway
Coronavirus disease—COVID-19	Coronavirus disease—COVID-19	TNF signalling pathway
Huntington disease	Oxidative phosphorylation	AMPK signalling pathway
Thermogenesis	Parkinson disease	p53 signalling pathway
Oxidative phosphorylation	Oocyte meiosis	PI3K-Akt signalling pathway
Endocytosis	Huntington disease	ABC transporters
Parkinson disease	Amyotrophic lateral sclerosis	Toxoplasmosis
Amyotrophic lateral sclerosis	Alzheimer disease	mTOR signalling pathway
Oocyte meiosis	Endocytosis	Small cell lung cancer
Virion—Herpesvirus	Prion disease	Focal adhesion

**Table 3:** Reactome pathway and interactor analysis for the comparisons between MST-H and mICSI-H blastocysts. The full list of pathways and interactions is available in Supplementary Table S5. Pathways and interactors highlighted in red are associated with the preimplantation embryo.

MST-H vs. mICSI-H	Directed Regulation
Antiviral mechanism by IFN-stimulated genes	Up
Interactions of Rev with host cellular proteins	Up
Nuclear import of Rev protein	Up
Nuclear Receptor transcription pathway	Up
CD28 dependent PI3K/Akt signalling	Up
RHO GTPases regulate CFTR trafficking	Up
Defective CFTR causes cystic fibrosis	Up
Deubiquitination	Up
Ub-specific processing proteases	Up
TP53 regulates transcription of additional cell cycle genes whose exact role in the p53 pathway remain uncertain	Up
PI5P, PP2A and IER3 Regulate PI3K/AKT Signalling	Up
Ethanol oxidation	Up
Interferon gamma signalling	Up
Cargo recognition for clathrin-mediated Endocytosis	Up
Transcriptional regulation by the AP-2 (TFAP2) family of transcription factors	Up
TFAP2 (AP-2) family regulates transcription of growth factors and their receptors	Up
RUNX1 regulates oestrogen receptor mediated transcription	Up
RUNX1 regulates transcription of genes involved in WNT signalling	Up
RHOQ GTPase cycle	Up
Defective OPLAH causes OPLAHD	Down
MGMT-mediated DNA damage reversal	Down

**Table 4:** Top 20 differentially expressed genes assessing the developmental potential of nuclear transfer through the comparison of MST hatching blastocysts with mICSI expanded blastocysts. The full list of genes is available in Supplementary Table S6. Genes highlighted in red are associated with the preimplantation embryo.

Gene ID	Gene or Human Equivalent	Function
CYR61	Cysteine-rich angiogenic inducer 61 (CCN1)	A promoter of cell adhesion, proliferation, and angiogenesis.
LOC100511133	Developmental pluripotency-associated protein 3-like	Transcript variant.
AASS	Alpha-aminoadipic semialdehyde synthase	Initiates the lysine degradation pathway in mammals.
ATP1B3	Sodium/potassium-transporting ATPase subunit beta-3	Regulates Na^+^ and K^+^ gradients in osmosis and electrical excitation.
BCAR1	Breast cancer anti-oestrogen resistance protein 1	Involved in cell motility, apoptosis, and cell cycle control.
OIP5	Opa Interacting Protein 5	Involved in packaging of telomere ends, cell cycle, and mitosis.
DLD	Dihydrolipoamide Dehydrogenase (mitochondrial)	Present in multi-enzyme complexes regulating energy metabolism; is also a protease in its monomeric form.
IQGAP2	IQ Motif Containing GTPase Activating Protein 2	Regulates cell morphology and motility, acts as a tumour suppressor, and is involved in antiviral responses.
DPPA3	Developmental pluripotency-associated protein 3	Represses transcription, regulates cell division and pluripotency.
SOAT1	Sterol O-Acyltransferase 1	An endoplasmic reticulum factor that catalyses fatty acid-cholesterol ester formation.
GK	Glycerol Kinase	Regulates uptake of glycerol and metabolism. It catalyses glycerol phosphorylation through ATP, producing ADP and glycerol-3-phosphate.
HENMT1	HEN Methyltransferase 1	Promotes small RNA 2′-O-methyltransferase in RNA methylation.
HNRNPA3	Heterogeneous Nuclear Ribonucleoprotein A3	Promotes RNA binding activity and mRNA splicing.
SSFA2	Sperm-specific antigen 2	Promotes actin filament binding in the cytosol nucleoplasm and plasma membrane.
LDHB	Lactate dehydrogenase B	Catalyses the interconversion of pyruvate and lactate.
ANP32E	Acidic Nuclear Phosphoprotein 32 Family Member E	Enables histone binding activity; histone chaperone activity; and protein folding chaperone.
IRS2	Insulin Receptor Substrate 2	Regulates insulin, insulin-like growth factor 1, through receptor tyrosine kinases and other downstream regulators.
CLU	Clusterin	A chaperone secreted under stress conditions in the cytosol. Involved in cell death, tumour onset, and neurodegenerative disorders.
ABCG1	ATP Binding Cassette Subfamily G Member 1	Involved in transport of macrophage cholesterol and phospholipids.
CRIP2	Cysteine Rich Protein 2	A potential transcription factor possessing two LIM zinc-binding domains. It may mediate smooth muscle differentiation.

**Table 5:** KEGG pathway analysis for differentially expressed genes from the comparison between MST-H and mICSI-E blastocysts. Only the top 10 pathways are shown. The full list of pathways is available in Supplementary Tables S2–S4. Pathways highlighted in red are associated with the preimplantation embryo.

MST-H vs. mICSI-E (Overall)	MST-H vs. mICSI-E (Upregulated)	MST-H vs. mICSI-E (Downregulated)
Oxidative phosphorylation	Oxidative phosphorylation	Pathways in cancer
Non-alcoholic fatty liver disease	Metabolic pathways	Human papillomavirus infection
Cell cycle	Cell cycle	Hippo signalling pathway
Diabetic cardiomyopathy	Non-alcoholic fatty liver disease	PI3K-Akt signalling pathway
Cellular senescence	Amyotrophic lateral sclerosis	ECM-receptor interaction
Amyotrophic lateral sclerosis	Citrate cycle (TCA cycle)	Focal adhesion
Chemical carcinogenesis—reactive oxygen species	Parkinson’s disease	Signalling pathways regulating pluripotency of stem cells
Parkinson disease	DNA replication	MicroRNAs in cancer
Citrate cycle (TCA cycle)	Diabetic cardiomyopathy	MAPK signalling pathway
Human papillomavirus infection	Carbon metabolism	Inflammatory bowel disease

**Table 6:** Reactome pathway and interactor analysis for the comparison between MST-H and mICSI-E blastocysts. The full list of pathways and interactions is available in Supplementary Table S7. Pathways and interactors highlighted in red are associated with the preimplantation embryo.

MST-H vs. mICSI-E	Directed Regulation
Cam-PDE 1 activation	Up
Classical Kir channels	Up
Aerobic respiration and respiratory electron transport	Up
Reverse Transcription of HIV RNA	Up
Minus-strand DNA synthesis	Up
Plus-strand DNA synthesis	Up
2-LTR circle formation	Up
Integration of viral DNA into host genomic DNA	Up
Autointegration results in viral DNA circles	Up
APOBEC3G mediated resistance to HIV-1 infection	Up
Calcineurin activates NFAT	Up
HDMs demethylate histones	Up
Defective MTRR causes HMAE	Up
Defective MTR causes HMAG	Up
Defective MMADHC causes MMAHCD	Up
Defective MMACHC causes MAHCC	Up
CD28 dependent PI3K/Akt signalling	Up
Alpha-oxidation of phytanate	Up
Defective ABCB11 causes PFIC2 and BRIC2	Up
Respiratory electron transport	Up
TICAM1 deficiency	Down

## Data Availability

Raw sequence data for gene expression are available under BioProjects PRJNA1139228 and 1380909.

## Supplementary Materials

The following supporting information can be downloaded at: https://www.mdpi.com/article/10.3390/epigenomes10020038/s1, Table S1. Differentially expressed genes at FDR < 0.05 for the comparison between MST-hatching (MST-H) blastocysts and mICSI-hatching (mICSI-H) blastocysts. Table S2. KEGG Pathway Analysis for all differentially expressed genes from the comparisons between MST-hatching (MST-H) blastocysts and mICSI-hatching (mICSI-H) blastocysts; and MST-hatching (MST-H) blastocysts and mICSI-expanded (mICSI-E) blastocysts. Table S3. KEGG Pathway Analysis for upregulated differentially expressed genes from the comparisons between MST-hatching (MST-H) blastocysts and mICSI-hatching (mICSI-H) blastocysts; and MST-hatching (MST-H) blastocysts and mICSI-expanded (mICSI-E) blastocysts. Table S4. KEGG Pathway Analysis for downregulated differentially expressed genes from the comparisons between MST-hatching (MST-H) blastocysts and mICSI-hatching (mICSI-H) blastocysts; and MST-hatching (MST-H) blastocysts and mICSI-expanded (mICSI-E) blastocysts. Table S5. Reactome Pathway Analysis for all differentially expressed genes from the comparison between MST-hatching (MST-H) blastocysts and mICSI-hatching (mICSI-H) blastocysts. Table S6. Differentially expressed genes at FDR < 0.05 for the comparison between MST-hatching (MST-H) blastocysts and mICSI-expanded (mICSI-E) blastocysts. Table S7. Reactome Pathway Analysis for all differentially expressed genes from the comparison between MST-hatching (MST-H) blastocysts and mICSI-expanded (mICSI-E) blastocysts. Table S8. Commonly and uniquely expressed genes for the differentially expressed genes at FDR < 0.05 for the comparisons between MST-hatching (MST-H) blastocysts and mICSI-hatching (mICSI-H) blastocysts; and MST-hatching (MST-H) blastocysts and mICSI-expanded (mICSI-E) blastocysts. Table S9. Reactome Pathway Analysis for the uniquely differentially expressed genes from the comparison between MST-hatching (MST-H) blastocysts and mICSI-hatching (mICSI-H) blastocysts. Table S10. Reactome Pathway Analysis for the uniquely differentially expressed genes from the comparison between MST-hatching (MST-H) blastocysts and mICSI-expanded (mICSI-E) blastocysts. Table S11: Media composition for Porcine Zygotic Media (PZM) and Porcine X Medium (PXM).
